# Psoralen Inhibited Apoptosis of Osteoporotic Osteoblasts by Modulating IRE1-ASK1-JNK Pathway

**DOI:** 10.1155/2017/3524307

**Published:** 2017-03-02

**Authors:** Shuqing Chen, Yongqian Wang, Yubin Yang, Ting Xiang, Jiahui Liu, Houming Zhou, Xinlin Wu

**Affiliations:** ^1^Department of Traditional Chinese Medicine, The First Affiliated Hospital of Sun Yat-sen University, Guangzhou 510080, China; ^2^Department of Musculoskeletal Oncology, The First Affiliated Hospital of Sun Yat-sen University, Guangzhou 510080, China

## Abstract

Osteoporosis is a common disease causing fracture in older populations. Abnormal apoptosis of osteoblasts contributes to the genesis of osteoporosis. Inhibiting apoptosis of osteoblasts provides a promising strategy to prevent osteoporosis. The proliferation of osteoblasts isolated from osteoporotic patients or healthy subjects was determined by MTT assay. Apoptosis was determined by Annexin V/PI assay. Protein expression was measured by western blot. The proliferation of osteoblasts isolated from osteoporotic patients was inhibited and the apoptosis level of these cells was higher than the osteoblasts from healthy subjects. Incubation with psoralen or estradiol significantly enhanced the proliferation and decreased the apoptosis level of osteoporotic osteoblasts. Western blot demonstrated that psoralen or estradiol treatment downregulated the expression of IRE1, p-ASK, p-JNK, and Bax. Meanwhile, expression of Bcl-2 was upregulated. Pretreatment by IRE1 agonist tunicamycin or JNK agonist anisomycin attenuated the effect of psoralen on osteoporotic osteoblasts. Psoralen inhibited apoptosis of osteoporotic osteoblasts by regulating IRE1-ASK1-JNK pathway.

## 1. Introduction

Osteoporosis is a disease which was characterized by low bone mass and increased susceptibility to fracture [1]. It is a major reason for fracture among older people [2]. Single vertebral fracture increased the risk of multiple vertebral fractures which impair patients' quality of life and increase mortality [3]. Although plenty of therapies were developed to prevent and treat osteoporosis and related fractures, there is still a lack of effective treatment for osteoporosis owing to limited understanding of its mechanism [4].

Pathogenesis of osteoporosis is a complex process. Age-related sex hormone deficiency and other factors result in the increased bone resorption but reduced the bone formation by decreasing the number and quality of osteoclasts. Therefore, insufficient bone formation relative to bone resorption plays an important role in mediating bone loss accompanied with aging and sex hormone deficiency [5]. Current evidence suggested that estrogen prevents bone loss owing to its antiapoptosis effects in osteoblasts, but the mechanism has not been fully investigated.

Traditional Chinese Medicine* Psoralea corylifolia* L. exhibited antitumor, antioxidant, antibacterial, antifungal immunomodulatory, estrogenic, and osteoblastic activities [6]. As the main active component of* Psoralea corylifolia* L., psoralen was reported possessing cartilage protecting [7], antitumor [8, 9], immunomodulatory [10], antivitiligo [11], and antiurticaria activities [12]. Its effect on osteoporosis and its mechanism were rarely reported.

In this study, we investigated the effect of psoralen on the osteoblast and underlying mechanism. Our results demonstrated that psoralen enhanced the proliferation of osteoblast and blocked the apoptosis of osteoblast by modulating inositol-requiring enzyme 1 (IRE1)/apoptosis signaling kinase 1 (ASK1)/c-jun N-terminal kinase (JNK) pathway.

## 2. Materials and Methods

### 2.1. Reagents

Psoralen, estradiol, IRE1 agonist tunicamycin (TM), and JNK agonist anisomycin (AM) were purchased from Sigma. Compounds were dissolved in DMSO to form stock solution and stored at 4°C.

### 2.2. Cell Culture

Osteoporotic osteoblasts were isolated from hip joint of postmenopausal osteoporotic women who were 50 to 55 years old [13]. Normal osteoblasts were obtained from the healthy postmenopausal women who were 50 to 55 years old. Cells were cultured in *α*-MEM medium (Gbico) supplemented with 10% fetal bovine serum (GENVIEW), 100 IU/mL penicillin, and 100 *μ*g/mL streptomycin at 37°C, 5% CO_2_, and a humidified atmosphere.

### 2.3. Cell Viability Assay

The proliferation of osteoporotic and normal osteoblasts and the effect of psoralen and estradiol (E2) on cell growth of osteoporotic osteoblasts were determined by 3-(4,5-dimethylthiazol-2-yl)-2,5-diphenyltetrazolium bromide (MTT) assay. Different osteoblasts were seeded at a density of 1 × 10^3^/well in 96-well plates and incubated at 37°C for 24 h. Cells were treated by psoralen (16 *μ*mol/L) or E2 (10 nmol/L) and then incubated for different time (0, 24, 36, 48, or 72 h). Then 20 *μ*L of 5 mg/mL MTT was added to each well. After 4 h incubation at 37°C, the supernatants were removed carefully and 150 *μ*L of DMSO was added. The optical density (OD) value was measured at 490 nm on Multiskan MK3 microplate reader (Thermo Fisher Scientific, USA). For the experiments using IRE1 agonist and JNK agonist, cells pretreated by TM or AM or DMSO for 1 h were incubated with psoralen for 48 h, and cell viability was determined following the standard procedure described above. Cell proliferation was assayed using EDU (RIBOBIO, Cat. No. C10327) according to the instruction of manufacture.

### 2.4. Apoptosis Analysis

Cell apoptosis was determined by flow cytometry using Annexin V/PI apoptosis kit (MultiSciences Biotech, China). Briefly, after pretreatment by TM (5 ug/mL), AM (1 ug/mL), or DMSO for 1 h and incubation with DMSO, psoralen (16 *μ*mol/L), or E2 (10 nmol/L) for 48 h, the cells were collected, washed with PBS, and resuspended in 0.5 mL 1x annexin-binding buffer at the density of 5 × 10^5^ cells/mL. Annexin V-FITC and PI were added and the cells were incubated for 10 min at room temperature. Samples were immediately analyzed by flow cytometry.

### 2.5. Real-Time PCR

Total RNA of each group was isolated by using total RNA isolation kit (Sangon Biotech Co., Ltd., China) according to corresponding manufacturer's protocol. Then the cDNA was synthesized using myoblastosis virus reverse transcriptase (AMV-RT) (Thermo Fisher Scientific, Inc., USA). In detail, the reactions were 10 min at 50°C and 10 min at 80°C, and then reaction system was cooled to 4°C. Specific primers used were as follows: GAPDH sense, 5′-TGTTCGTCATGGGTGTGAAC-3′ and antisense, 5′-ATGGCATGGACTGTGGTCAT-3′; IRE1 sense, 5′-GCAAGAGTATGTGGAGCAGAAGG-3′ and antisense, 5′-TGTGAACGATGTTGAGGGAGTG-3′; Bax sense, 5′-ATCATGGGCTGGACATTGGA-3′ and antisense, 5′-ACAGGGACATCAGTCGCTTCA-3′; Bcl-2 sense, 5′-GGGAGGATTGTGGCCTTCTT-3′ and antisense, 5′-TGTGCAGGTGCCGGTTCAG-3′. Subsequently, qPCR was conducted and signals were collected by using Stratagene Mx3000P real-time PCR (Agilent, USA). qPCR assays were performed at followed reaction system: 2 min at 94°C, 20 sec at 94°C, then 59°C for 20 sec, and, finally, 40 cycles of 72°C for 20 sec. Data analysis was performed using the 2^−ΔΔCt^ method. Every experiment was carried out three times.

### 2.6. Western Blot

The effect of psoralen, E2, psoralen + TM, and psoralen + AM on the expression of different proteins was determined by western blot. After pretreatment by TM (5 ug/mL), AM (1 ug/mL), or DMSO for 1 h and incubation with DMSO, psoralen (16 *μ*mol/L), or E2 (10 nmol/L) for 48 h, cells were washed with PBS and lysed. By centrifugation for 10 min at 14000 rpm under 4°C, the supernatants were collected and the protein concentration was determined by Pierce™ BCA Protein Assay Kit (Thermo, USA). Equal amounts of protein (20 *μ*g) were separated by SDS-PAGE and transferred to PVDF membranes (Millipore). After being washed with TBS, the membranes were blocked with TBS containing 5% skim milk and incubated with primary antibodies. After being washed with TBST, the membranes were incubated with HRP-conjugated secondary antibody (HRP Goat anti-Rabbit IgG, BOSTER). Then the membranes were developed with electrochemiluminescence system. Finally, the densitometry of protein bands was measured using Image-Pro Plus 6.0 system and normalized to relevant controls.

### 2.7. Statistical Analysis

Student's *t*-test or one-way ANOVA was used to compare the differences among groups. *P* < 0.05 was considered statistically significant. Then data were analyzed using Graphpad Prism 5.

## 3. Results

### 3.1. Proliferation of Primary Osteoblasts Isolated from Osteoporotic Patients Was Inhibited

**Figure 1:**
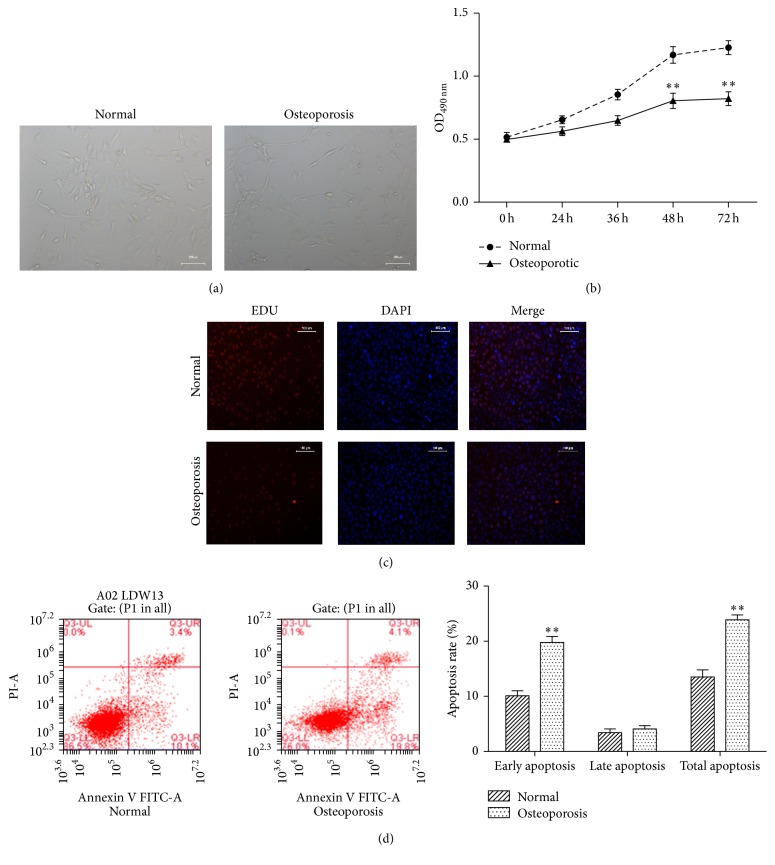
Proliferation of primary osteoblasts isolated from osteoporotic patients was inhibited. (a) Optical microscopic observation of osteoblasts from normal and osteoporotic subjects after 72 h incubation. (b) Proliferation of osteoblasts from osteoporotic patients was inhibited. Osteoporotic and normal osteoblasts were incubated for 72 h. The cell viability at different time points was determined by MTT assay. (c) The EDU stain also performed in cells treated as described above with a magnification of 200. (d) Osteoporotic osteoblasts exhibited higher apoptosis level. Apoptosis was determined by Annexin V/PI assay. ^*∗∗*^*P* < 0.01 compared with normal group.

To investigate the different cell behavior between osteoporotic osteoblasts and normal osteoblasts, we collected osteoblasts from osteoporotic patients and healthy subjects. The proliferation of osteoblasts was determined at different time points. As shown in Figures 1(a), 1(b), and 1(c), the proliferation of osteoblasts from osteoporotic patients was significantly inhibited compared with normal osteoblasts after 48 h incubation. Then apoptosis levels of two sources of osteoblasts were determined. It was discovered that more osteoporotic osteoblasts induced early apoptosis (Figure 1(d)).

### 3.2. Psoralen Increased the Proliferation and Inhibited the Apoptosis of Osteoporotic Osteoblasts

**Figure 2:**
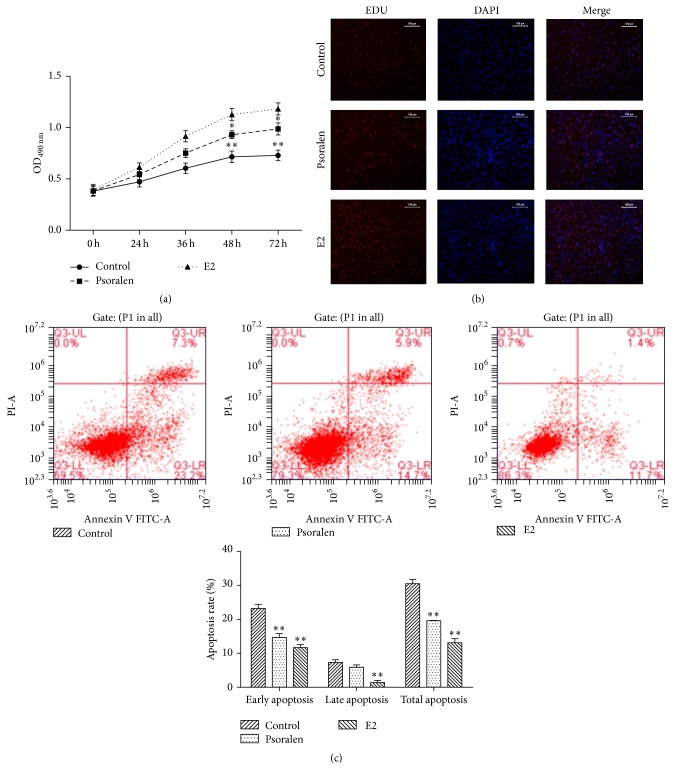
Psoralen increased the proliferation and inhibited the apoptosis of osteoporotic osteoblasts. (a) Psoralen increased the proliferation of osteoporotic osteoblasts. Osteoporotic osteoblasts treated by DMSO, psoralen, and E2 were incubated for 72 h. The cell viability at different time points was determined by MTT assay. (b) The EDU stain also performed in cells treated as described above with a magnification of 200. (c) Psoralen inhibited the apoptosis of osteoporotic osteoblasts. Osteoporotic osteoblasts were coincubated with DMSO, psoralen, or E2 for 48 h. Then cell apoptosis was determined by Annexin V/PI assay. ^*∗*^*P* < 0.05, ^*∗∗*^*P* < 0.01 compared with control.

To investigate the effect of psoralen on the osteoporosis, osteoporotic osteoblasts were treated by psoralen. E2 was used as positive control. As shown in Figures 2(a) and 2(b), psoralen treatment significantly enhanced the proliferation of osteoporotic osteoblasts compared with control, but this effect was weaker than E2. Flow cytometry analysis demonstrated that the apoptosis levels of osteoporotic osteoblasts were decreased after treatment by psoralen and E2 (Figure 2(c)).

### 3.3. Psoralen Regulated the IRE1-ASK1-JNK Pathway

**Figure 3:**
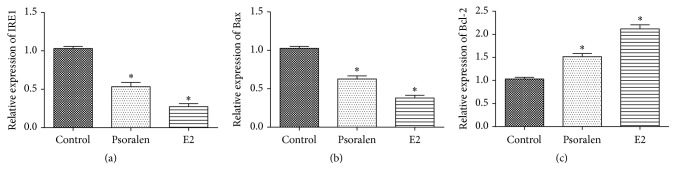
Psoralen regulated the mRNA expression of IRE1, Bax, and Bcl-2. (a, b, and c) Osteoporotic osteoblasts were pretreated by psoralen or E2 for 48 h, and then the mRNA expressions of IRE1, Bax, and Bcl-2 were assayed by qPCR. ^*∗*^*P* < 0.05 versus control group.

**Figure 4:**
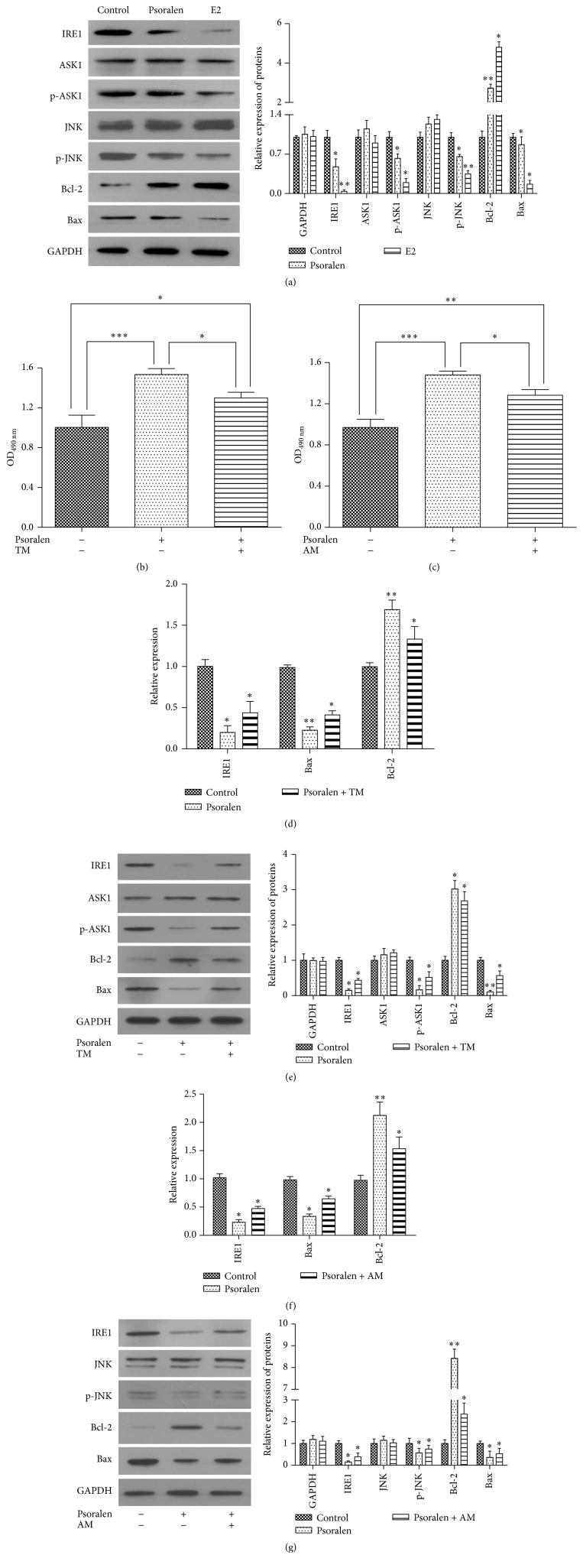
Psoralen regulated the IRE1-ASK1-JNK pathway in osteoporotic osteoblasts. (a, e, and g) Osteoporotic osteoblasts were pretreated by TM, AM, or DMSO for 1 h, and then osteoblasts were incubated with psoralen or E2 for 48 h. Then protein expression was determined by western blot, and (d and f) mRNA expression was determined by qPCR. (b and c) Osteoporotic osteoblasts were pretreated by TM or AM for 1 h. Then the cells were incubated with psoralen for 48 h. The cell viability was determined by MTT assay. ^*∗*^*P* < 0.05, ^*∗∗*^*P* < 0.01, ^*∗∗∗*^*P* < 0.001.

In order to explore the mechanism of protective effects of psoralen and E2 on osteoporotic osteoblasts, we determined the expression of proteins in IRE1-ASK1-JNK cascade. As shown in Figures 3(a) and 4(a), psoralen and E2 treatment dramatically decreased the expression of upstream IRE1. They did not influence the expression of ASK1 and JNK but reduced the phosphorylation of ASK1 and JNK. Moreover, the mRNA and protein expression of Bcl-2 was enhanced and expression of Bax was decreased (Figures 3(b), 3(c), and 4(a)).

To validate the effect of psoralen on the IRE1-ASK1-JNK pathway, osteoblasts were pretreated by IRE agonist TM and JNK agonist AM. When osteoporotic osteoblasts were pretreated by TM for 1 h and then incubated with psoralen for 48 h, decreased expression of IRE1, p-ASK1, and Bax and increased expression of Bcl-2 induced by psoralen alone were attenuated in both mRNA and protein expression (Figures 4(d) and 4(e)). Cell growth enhanced by psoralen was also decreased (Figure 4(b)).

Similarly, cotreatment of psoralen with AM also reversed the effect of psoralen on the mRNA and protein expression of p-JNK, Bcl-2, and Bax (Figures 4(f) and 4(g)) and cell proliferation (Figure 4(c)).

These results together demonstrated that psoralen inhibited the apoptosis of osteoporotic osteoblasts by adjusting the IRE1-ASK1-JNK pathway.

## 4. Discussion

Although there was report indicating that idiopathic osteoporotic and normal cells had no differences in their growth rate [14], more studies supported our results that osteoporotic osteoblasts exhibited inhibitory cell growth compared with normal cell. Ruiz-Gaspa et al. found that osteoblast derived from the patients with male idiopathic osteoporosis had slower proliferation rate [15]. Battmann et al. also reported that growth of endosteal bone cells was impaired in men aged over 50 years, but not in women and men aged under 40 years [16]. These results indicated that this functional loss in proliferation was associated with age and gender of patients and probably the subtypes of osteoporosis. Further studies should be performed to identify the differences and elucidate the mechanism.

Apoptosis is the fate of majority of osteoblasts. We discovered that the lower growth rate was associated with higher apoptotic level of osteoporotic osteoblasts, which could be reversed by E2 and psoralen.

The efficacy of estrogen in combating osteoporosis was well demonstrated by large-scale clinical trials and meta-analysis [17, 18]. Estrogen exhibited its osteoprotective activity by remodeling units and unbalanced coupling of bone resorption and formation. The key mechanism of estrogen's effects is through regulation of osteoblast longevity by inhibition of osteoblast apoptosis. But the signal pathway by which estrogen inhibits osteoblast apoptosis was not fully understood. E2 affected caspase-3/7 activity in G-292 cell model and significantly inhibited the apoptosis-related gene expression (BID, LTBR, TNFRSF-12, RIP2, and InsP3R) [19]. Recent study also reported that 17beta-estradiol inhibited the ER stress-induced apoptosis by promoting Ras-ERK1/2-TFII-I signaling pathway-dependent Grp78 induction in MC3T3-E1 cells [20], which reminded us that endoplasmic reticulum induced IRE1-ASK1-JNK apoptosis pathway may also be involved in the E2 effect on osteoblast. In this study, we demonstrated that E2 can inhibit the apoptosis by modulating IRE1-ASK1-JNK pathway, which may provide a new target for treating osteoporosis.

Psoralen was a natural product possessing cartilage protecting [7], antitumor [8, 9], immunomodulatory [10], antivitiligo [11], and antiurticaria activities [12]. It was commonly used in combination with UVA to form a phototherapy, called psoralen plus ultraviolet A (PUVA), to treat cancers. In human hepatocarcinoma SMMC-7721 cells, psoralen induced the cell apoptosis via adjusting caspase-3, p53, Bax, and Bcl-2 pathway [21]. PUVA also reduced p85(ErbB2) phosphorylation to induce apoptosis of ErbB2+ breast cancer models [22]. Activation of platelet-activating factor (PAF) pathway is crucial for PUVA-induced immune suppression, skin inflammation, and apoptosis [23]. Fas-FasL and p53 pathway regulation was also involved in the process of apoptosis of mouse epidermal cells induced by PUVA treatment [24]. There were two studies that investigated the effect of psoralen on osteoporosis. One study demonstrated that psoralen could promote osteoblast differentiation through the activation of BMP signaling in primary mouse calvarial osteoblasts in vitro [25]. Another study described that psoralen promoted the bone mass in ovariectomy-induced osteoporotic rats by stimulation of differentiation of bone mesenchymal stem cell to osteoblasts [26]. In this study, we found that psoralen exhibited similar effect to E2 that inhibited the apoptosis by modulating IRE1-ASK1-JNK pathway. Psoralen provided a promising therapy for the treatment of osteoporosis.

In conclusion, psoralen inhibited the apoptosis and increased the proliferation of osteoporotic osteoblasts by modulating IRE1-ASK1-JNK pathway. Targeting IRE1-ASK1-JNK pathway may provide a good strategy for developing effective therapies for osteoporosis.
